# Effects of Aging on Pain Tolerance: A Comparative Study of Heat Pain Thresholds and Tonic Heat and Cold Stimuli

**DOI:** 10.1002/ejp.70180

**Published:** 2025-12-02

**Authors:** Julia Devanne, Louise Trocmet

**Affiliations:** ^1^ Centre National de la Recherche Scientifique, Laboratoire de Neurosciences Cognitives et Adaptatives Université de Strasbourg Strasbourg France; ^2^ INSERM, Imaging Brain & Neuropsychiatry iBraiN U1253 Université de Tours Tours France

**Keywords:** aging, pain threshold, pain tolerance, thermal pain

## Abstract

**Introduction:**

Aging is known to impact both pain perception and modulation. While sensory thresholds have been extensively studied, age‐related changes in pain tolerance remain less conclusive. Additionally, limited research has explored whether thermal modality differentially affects pain tolerance in older adults. This study aimed to examine modality‐specific effects of aging on pain tolerance using three experimental paradigms: thermal pain tolerance thresholds, and tolerance to tonic heat and cold stimulation.

**Methods:**

Forty‐two healthy participants were recruited. Heat pain tolerance thresholds were assessed using a contact thermode. Tonic pain tolerance was measured by exposing the hand to heat or cold air for up to 10 min in a hermetically sealed box. Outcome measures included tolerance duration, pain onset latency, and subjective pain ratings.

**Results:**

No significant age‐related differences were observed in heat pain tolerance thresholds. However, older adults exhibited significantly reduced tolerance to prolonged heat stimulation, with shorter tolerance durations and higher pain ratings. In contrast, pain responses in the cold condition did not differ between age groups.

**Conclusion:**

Aging appears to selectively reduce tolerance to sustained heat stimulation, whereas tolerance to cold and brief stimuli is relatively preserved. This supports the existence of modality‐specific changes in pain processing across the lifespan.

**Significance:**

These results underscore the importance of stimulation characteristics in evaluating pain tolerance across the lifespan. The study highlights an underexplored heat‐specific vulnerability in older adults, offering insights for refining experimental pain models, improving age‐sensitive assessments, and encouraging further research into physiological mechanisms underlying altered pain processing in aging.

## 1 Introduction

Aging is associated with well‐documented structural and functional changes in the somatosensory system that influence pain perception and modulation. Among these alterations, the integrity of nociceptive processing has gained increasing attention, given the elevated risk of chronic pain in older adults. Epidemiological data show that approximately 30% of adults over 18 report chronic pain, rising to over 50% beyond age 60 (Gagliese 2009; Patel et al. 2013). These figures highlight the need to better understand how aging affects pain sensitivity and tolerance.

While numerous studies have shown increased sensory and pain detection thresholds with age, indicating age‐related hypoesthesia (Edwards and Fillingim 2001; Jensen et al. 1992; Lautenbacher et al. 2005; Magerl et al. 2010; Marini et al. 2012; Pickering et al. 2002; Washington et al. 2000), evidence regarding pain tolerance remains inconsistent. Defined as the maximum intensity of a painful stimulus one is willing to endure, pain tolerance has produced divergent findings (Edwards and Fillingim 2001; Neziri et al. 2011; Pickering et al. 2002; Woodrow et al. 1972). Some studies report reduced tolerance in older adults (Petrini et al. 2015; Zhou et al. 2015), while others find no significant age effect (Neri and Agazzani 1984; Neziri et al. 2011; Washington et al. 2000).

This heterogeneity may stem in part from methodological variation, including differences in stimulus type and paradigm. Studies have used thermal (Edwards et al. 2003; Zhou et al. 2020), mechanical (Bek et al. 2002; Petrini et al. 2015), and electrical stimuli (Neri and Agazzani 1984), often yielding inconsistent outcomes. A meta‐analysis by Lautenbacher (2012) initially suggested an age‐related decline in pain tolerance, especially for thermal and mechanical stimuli. However, after adjusting for the disproportionate influence of a single large‐sample study (Woodrow et al. 1972; Lautenbacher et al. 2017) reported minimal effects, challenging the robustness of prior conclusions.

Tonic pain paradigms, which involve sustained nociceptive input, may offer better ecological validity than brief threshold tests by more closely approximating chronic pain experiences (Rainville et al. 1992; Mitchell et al. 2004; Edens and Gil 1995). Nonetheless, relatively few studies have explored this approach. Of the four studies examining tolerance to tonic cold pain, only two found reductions in older adults (Edwards et al. 2003; Neziri et al. 2011; Walsh et al. 1989; Washington et al. 2000). In contrast, the two available studies on prolonged heat stimulation both reported significant age‐related declines (Zhou et al. 2020; Devanne et al. 2024).

Thermal modality appears to be a key factor, as heat and cold pain involve partially distinct neurophysiological pathways and may be differentially affected by age. However, direct comparisons of modality effects in aging populations remain limited.

The present study aims to assess age‐related differences in pain tolerance by comparing responses to matched tonic heat and cold stimuli, and by evaluating how these differ from thermal pain tolerance thresholds. This design allows us to investigate whether aging confers a modality‐specific vulnerability to prolonged nociceptive input.

## 2 Materials and Methods

### 2.1 Participants

Twenty‐three young adults (11 men, 12 women; mean age: 21.7 ± 1.7 years) and 19 older adults (9 men, 11 women; mean age: 68.6 ± 4.6 years) were recruited for this study. Participant eligibility was determined through a detailed medical questionnaire aimed at identifying any conditions that might interfere with pain perception. All individuals were recruited from our laboratory database and were excluded if they reported a history of oncological conditions (tumours or cancer); neurological or neurodegenerative disorders (e.g., epilepsy, multiple sclerosis, Parkinson's disease), including central neurological diseases, peripheral nerve lesions, radiculopathy, or polyneuropathy; cardiovascular diseases (e.g., coronary heart disease, arrhythmia); gastrointestinal disorders (e.g., Crohn's disease, ulcers); liver or pancreatic diseases (e.g., hepatitis); metabolic or endocrine disorders (e.g., diabetes, thyroid dysfunction); musculoskeletal disorders (e.g., arthritis, osteoporosis); mental health disorders (e.g., depression, anxiety, etc.); pain‐related conditions such as migraine; or dermatological diseases (e.g., eczema, psoriasis).

In addition, participants did not take any medication that could interfere with thermal or pain perception at the time of testing or in the 6 months prior to the study. This included antidepressants, antiepileptics, psychostimulants, triptans, psycho–neuropharmacological agents, anticoagulants, or beta‐blockers. Moreover, no participant had taken any analgesic medication (including paracetamol/acetaminophen, acetylsalicylic acid, or other NSAIDs) during the 6 weeks preceding the experiment.

It should be noted that no clinical neurological examination was performed to screen for subclinical polyneuropathy.

Moreover, because a link between psychological factors such as anxiety and fear of pain could explain inter‐individual variability in pain perception, participants completed the State**–**Trait Anxiety Inventory (STAI) (Spielberger et al. 1983) and the Fear of Pain Questionnaire (McNeil and Rainwater 1998) at the beginning of the session.

All experimental procedures were approved by the local ethics committee and adhered to the principles outlined in the Declaration of Helsinki (accreditation number: Unistra/CER/2021‐05). Participants were provided with detailed information about the study protocol and gave written informed consent prior to participation. At the conclusion of the study, all participants were fully debriefed and compensated financially for their participation.

Demographic data and scores on psychological questionnaires are reported in Table 1.

**TABLE 1:** Demographic data and psychological questionnaire scores in the young and older groups.

	Young adults	Older adults	Group effect^a^	
			*U*	*p*
Number of subjects	23	19		
Mean age (SD)	21.7 (1.7)	68.6 (4.6)		
Proportion of males, %	47.8	47.4		
Mean score fear of pain questionnaire (SD)	41.9 (10.9)	37.65 (11.6)	173	0.17
Mean score STAI‐A (SD)	29.8 (5.7)	28.1 (7.3)	215	0.91
Mean score STAI‐B (SD)	41.9 (10.9)	35.5 (8.2)	228	0.97

Abbreviation: SD, standard deviation.

^a^ The effect of the group was analysed using a Mann–Whitney.

### 2.2 Procedure

Participants were comfortably seated in a room maintained at a temperature of 21°C (±2°C).

For each participant, we assessed heat pain tolerance thresholds, as well as tolerance to tonic pain induced by heat and cold. A 20‐min interval was maintained between measurements to ensure that any potential endogenous pain inhibition mechanisms had ceased.

To minimise concentration‐related variability during the experimental session, we counterbalanced the order of test administration among subjects.

#### 2.2.1 Heat Pain Tolerance Threshold

Pain tolerance thresholds were measured on the back of the participants' non‐dominant hand using a thermal stimulator (TCS II; QST.Lab, Strasbourg, France), which consists of a contact thermode and a control unit. The stimulator surface is composed of 15 Peltier elements, arranged in five zones of 7 × 12 mm each, with a total surface area of 4.2 cm^2^. The temperature is controlled with a relative precision of 0.1°C and an absolute precision of 0.5°C.

Tolerance thresholds were determined using the method of limits, which involves gradually increasing the temperature until the participant experiences intolerable pain, at which point they end the stimulation by pressing a response button. Prior to each trial, the thermode temperature was adjusted to match the participant's skin temperature (±0.1°C). The temperature then increased at a rate of 1°C per second from this baseline. Participants were instructed to press the response button when the pain became unbearable, which triggered an immediate return to baseline temperature at a rate of 170°C per second.

Each participant completed ten consecutive trials, with the thermode slightly repositioned between trials to minimise habituation or sensitization effects. The pain tolerance threshold was defined as the average temperature recorded across all trials.

#### 2.2.2 Tonic Pain Tolerance

##### 2.2.2.1 Tonic Cold Pain

An experimental method similar to the Cold Pressor Test (CPT) was employed, involving exposure to cold air within a hermetically sealed box. The air temperature was controlled by Peltier modules, maintaining a stimulation temperature with a relative precision of 0.5°C. Unlike the CPT, this model has the advantage of preventing the activation of cutaneous tactile receptors, thus providing a more specific stimulation of thermoreceptors. The air inside the box was kept at a temperature of 0°C (±0.5°C).

##### 2.2.2.2 Tonic Heat Pain

A similar setup was used to assess tolerance to tonic heat‐induced pain. The air inside the hermetically sealed box was maintained at a temperature of 70°C (±0.5°C). Participants were informed that the air inside the box would be very hot but would not cause any burns or skin damage.

Safety was ensured by continuous real‐time monitoring of chamber temperature, which never exceeded the 70°C target. Preliminary trials confirmed that skin temperature, measured with thermocouples, averaged 44°C, a value well below the thresholds for epidermal damage described by Moritz and Henriques (1947), thus validating the safety and reliability of the paradigm (Moritz and Henriques 1947).

In both conditions (i.e., tonic heat and cold pain), participants were instructed to place their hand in the box, keeping it open and still, while focusing on the sensations they perceived. They were asked to endure the pain for as long as possible, until the sensation became intolerable. At that point, they could end the test by withdrawing their hand from the box. If the pain sensation did not become unbearable within 10 min, the test was terminated. To account for the potential effect of manual laterality on pain perception, the exposure of the right and left hand was counterbalanced across participants.

Two objective behavioural measures were collected: (i) pain onset, defined as the time at which participants verbally reported the first perception of pain, and (ii) tolerance duration, defined as the total exposure time until hand withdrawal. In addition, two subjective ratings were obtained at the end of each test: (i) pain intensity, indexing the sensory–discriminative dimension of pain, rated on a numerical scale from 0 (“no pain sensation”) to 10 (“unbearable pain”), and (ii) pain unpleasantness, indexing the affective dimension, rated on a numerical scale from 0 (“no unpleasantness”) to 10 (“extremely unpleasant”).

The use of tonic warm and cold air stimulation was chosen to ensure strict comparability between the two modalities. The choice of a tonic heat paradigm is supported by previous work demonstrating its safety, reproducibility, and suitability for mechanistic investigations of prolonged pain (Naert et al. 2008). By contrast, the Cold Pressor Test (CPT), widely used as a reference for cold pain tolerance, is known to induce strong cardiovascular thermoregulatory responses (e.g., blood pressure increase, sympathetic activation) that may confound nociceptive responses (Korhonen 2006; Streff et al. 2010; von Baeyer et al. 2005). Also, it may selectively engage thermoreceptors while avoiding mechanical confounds such as hydrostatic pressure present in the CPT.

### 2.3 Statistical Analyses

Statistical analyses were performed using Statistica software (Softonic International, Barcelona, Spain). The normality of the data distributions was assessed using the Kolmogorov–Smirnov test. Among all variables, only pain tolerance thresholds followed a normal distribution.

Therefore, a one‐way ANOVA was conducted to assess the effect of age on pain tolerance thresholds.

For tonic heat and cold pain tests, non‐parametric Mann–Whitney *U* tests were used to evaluate the effect of age on tolerance duration, time of pain onset, and ratings of pain intensity and unpleasantness, due to non‐normal data distributions.

To assess the effect of thermal modality (heat vs. cold) on pain measures (tolerance duration, pain onset, pain intensity, and unpleasantness), Wilcoxon signed‐rank tests were carried out within each age group.

The alpha level was set at 0.05 for all statistical tests.

## 3 Results

Baseline skin temperature was assessed prior to experimental stimulation to control for potential physiological differences between groups. No significant difference was found between young adults (mean = 30.82°C, SD = 0.92) and older adults (mean = 30.4°C, SD = 1.54; *U* = 183.5, *p* = 0.26).

### 3.1 Pain Tolerance Threshold

**FIGURE 1:**
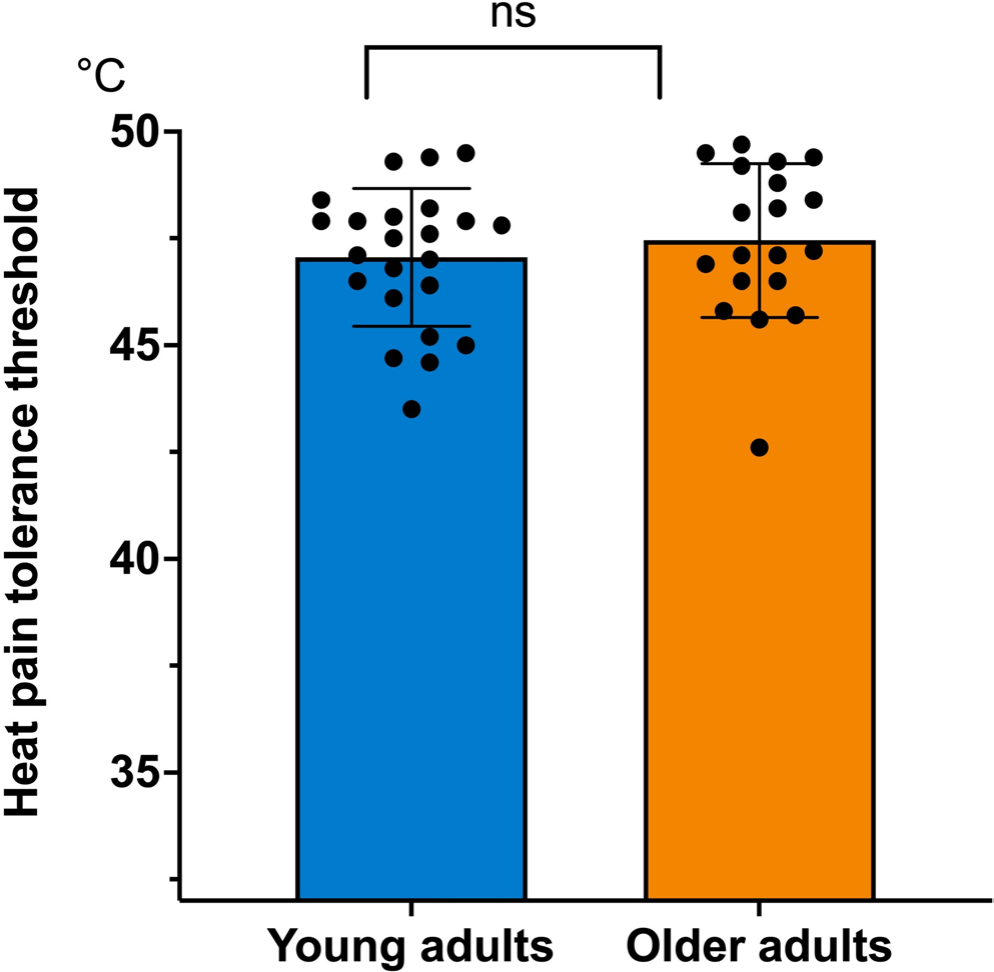
Heat pain tolerance threshold (in °C). ns = not significant.

No significant difference in heat pain tolerance thresholds was found between the two age groups (*F*(1, 40) = 0.56, *p* = 0.46) (young participants: mean = 47.1; SD = 1.6/older adults: mean = 47.45; SD = 1.8; Figure 1).

To account for potential habituation or anticipation effects, we compared tolerance thresholds between the first three and the last three trials using repeated‐measures ANOVAs. In young adults, no significant effect of trial block was observed (first three trials: mean = 47.2°C, SD = 1.5; last three trials: mean = 47.0°C, SD = 1.6; *F*(1, 22) = 0.49, *p* = 0.49). Similarly, in older adults, mean tolerance was 47.5°C (SD = 1.8) for the first three trials and 47.4°C (SD = 1.7) for the last three (*F*(1, 18) = 0.36, *p* = 0.56).

### 3.2 Tolerance to Tonic Heat Pain

It is important to note that two participants (one young and one older) did not report any pain sensation. Consequently, these two participants were excluded from the analyses related to pain onset.

**FIGURE 2:**
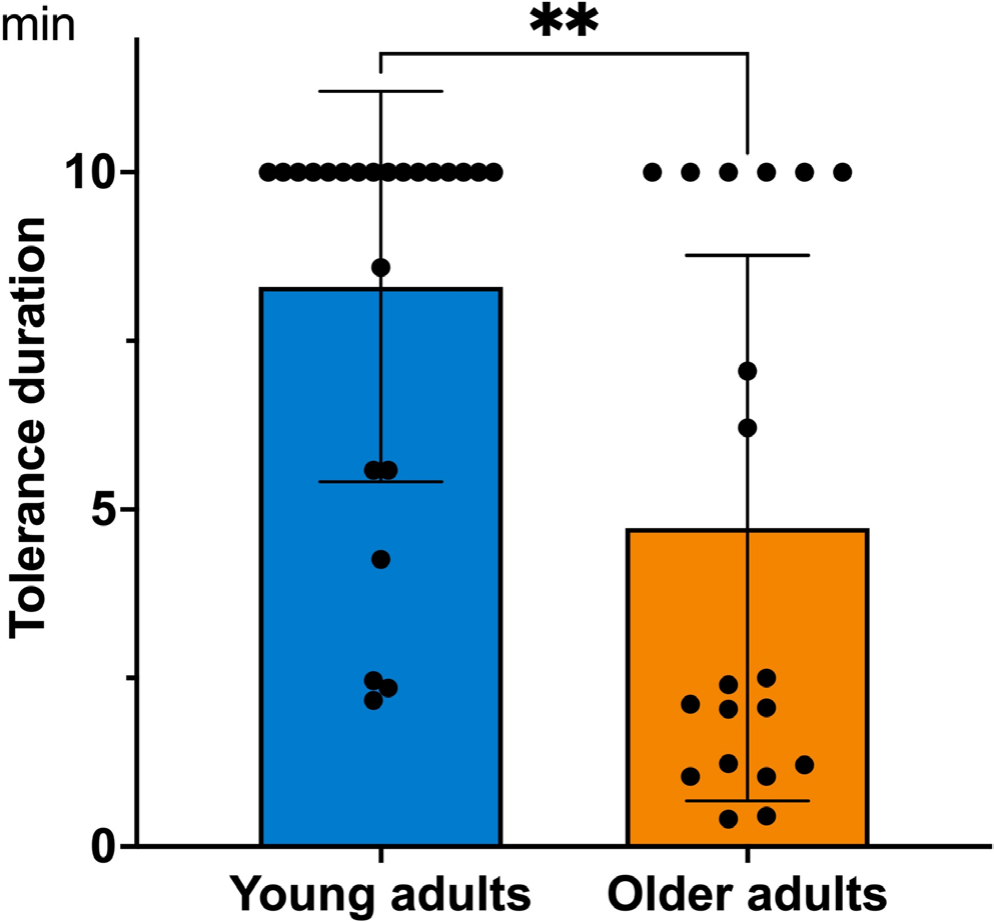
Tolerance duration to tonic heat pain (in minutes). **p < 0.01.

The results revealed an effect of age on the duration of tolerance to prolonged heat‐induced pain. Older adults exhibited a shorter tolerance duration (mean = 4.49 min; SD = 3.59 min) compared to younger participants (mean = 8.23 min; SD = 2.50 min; *U* = 107; *p* = 0.005; Figure 2). Moreover, 70% of young participants tolerated the pain until the end of the test, whereas this proportion was only 32% among older adults (*χ* ^2^ = 6.02; *p* = 0.01).

No significant effect was found regarding the onset of first pain between younger (mean = 1.24 min; SD = 0.46 min) and older participants (mean = 1.24 min; SD = 1.22 min) (*U* = 177; *p* = 0.4).

Analyses of the subjective ratings showed a trend toward an age effect for both the affective dimension (unpleasantness: *U* = 149.5, *p* = 0.08) and the sensory–discriminative dimension (pain intensity: *U* = 150, *p* = 0.08), with a higher tendency to report elevated ratings in older adults (Table 2).

**TABLE 2:** Effect of age group on perceived unpleasantness and pain intensity ratings during tonic heat and cold pain.

	Young adults		Older adults		Group effect^a^	
	Mean	SD	Mean	SD	*U*	*p*
*Tonic heat pain*						
Perceived unpleasantness ratings (0–10)	5.11	3.22	6.84	2.19	149.5	0.08
Perceived pain intensity ratings (0–10)	3.65	3.21	5.47	3.59	150	0.08
*Tonic cold pain*						
Perceived unpleasantness ratings (0–10)	5.76	2.90	4.87	3.07	187	0.43
Perceived pain intensity ratings (0–10)	3.96	2.89	2.82	3.17	165	0.18

Abbreviation: SD, standard deviation.

^a^ The effect of the group was analysed using a Mann–Whitney.

### 3.3 Tolerance to Tonic Cold Pain

Four older participants and 1 young adult did not perceive pain.

**FIGURE 3:**
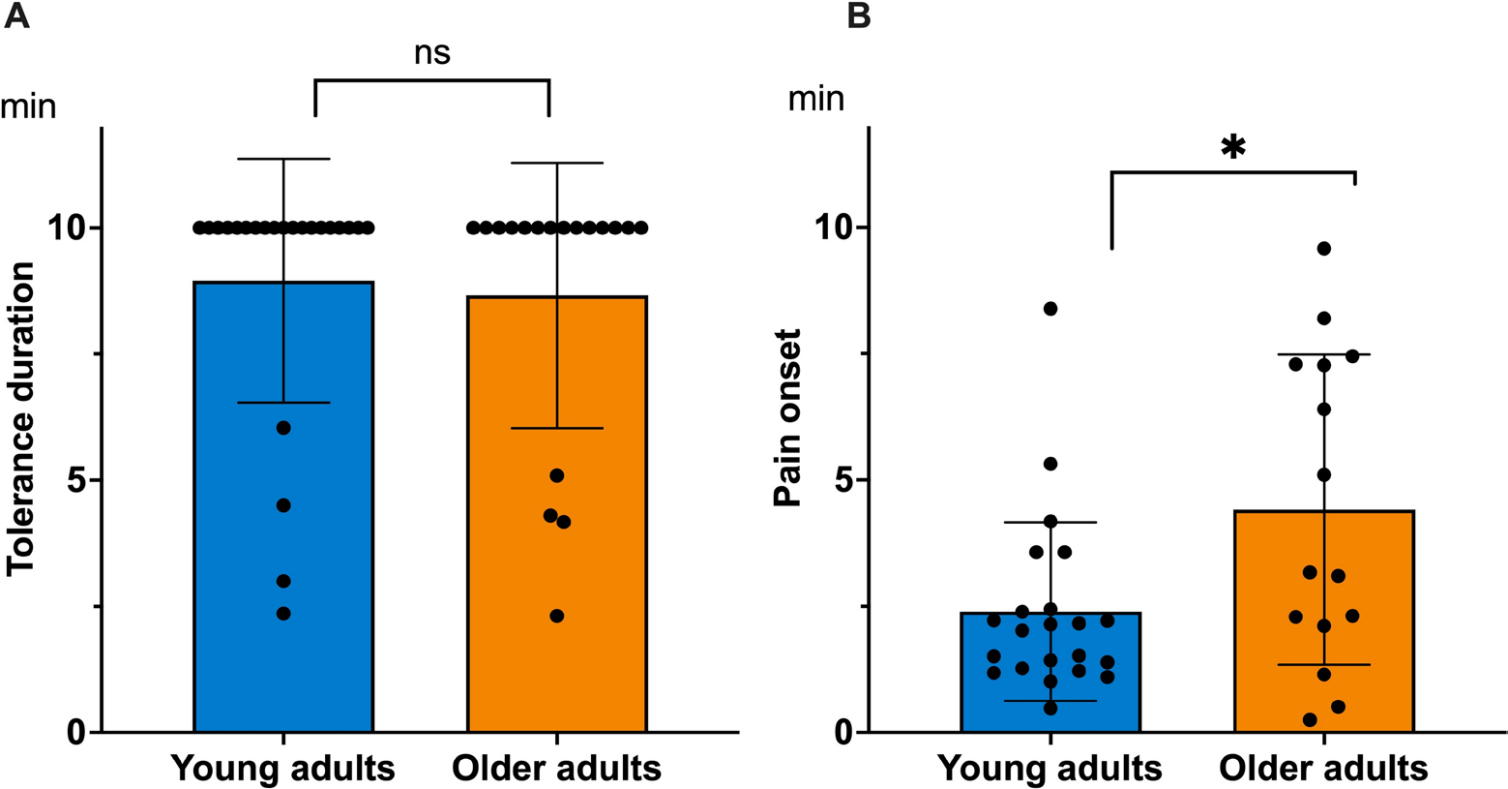
Tolerance duration to tonic cold pain (in minutes) (A) and onset of first pain (in minutes) (B) between the two age groups. *p < 0.05; ns, not significant.

The results showed no effect of age on tonic cold pain tolerance duration (*U* = 217; *p* = 0.98; Figure 3A). The mean tolerance duration was 8.48 min (±2.25 min) for younger participants and 8.45 min (±2.30 min) for older adults. Additionally, the proportion of participants who endured the test until the end did not differ between the two groups, with 79% in the older group and 78% in the younger group (*χ* ^2^ = 0; *p* = 0.96).

However, a significant age effect was observed on the onset of first pain, which appeared earlier in younger participants (mean = 2.36 min; SD = 1.48 min) compared to older adults (mean = 4.55 min; SD = 3.15 min; *U* = 108; *p* = 0.05; Figure 3B).

Analyses of the subjective ratings revealed no significant age effect for either the affective dimension (unpleasantness: *U* = 187, *p* = 0.43) or the sensory–discriminative dimension (pain intensity: *U* = 165, *p* = 0.18; Table 2).

### 3.4 Effect of Thermal Modality on Pain Measures

In young participants, thermal modality did not significantly influence the objective behavioural measures of pain tolerance duration (*T* = 6; *p* = 0.18) or pain onset, and it had no significant effect on the subjective ratings reflecting the sensory–discriminative (pain intensity; *T* = 93.5; *p* = 0.67) or affective (unpleasantness; *T* = 81; *p* = 0.57) dimensions. The only significant effect was observed for pain onset, which occurred earlier during heat compared to cold stimulation (*T* = 11; *p* < 0.001).

In older participants, thermal modality exerted a significant effect across both objective and subjective measures. Compared with cold stimulation, tonic heat stimulation resulted in reduced tolerance durations (*T* = 0; *p* < 0.001) and earlier pain onset (*F*(1, 15) = 25; *p* < 0.001), as well as higher sensory–discriminative ratings of pain intensity (*T* = 9; *p* = 0.01) and a trend toward higher affective ratings of unpleasantness (*T* = 33; *p* = 0.07).

## 4 Discussion

The objective of this study was to assess the impact of aging on pain tolerance using three experimental paradigms: thermal pain tolerance thresholds, and tonic pain assessments under heat and cold stimulation.

Importantly, our study combined both objective behavioural measures (pain onset latency and tolerance duration) and subjective ratings (pain intensity and unpleasantness). This multidimensional approach allowed us to examine how the type of stimulation (tonic vs. phasic) and the thermal modality (heat vs. cold) influence pain tolerance in older adults, while also distinguishing sensory‐discriminative and affective components of pain perception.

The present study demonstrates that aging does not uniformly diminish pain tolerance but instead exerts selective effects based on stimulus modality and duration. While no differences were observed between young and older adults in terms of heat pain tolerance thresholds, suggesting preserved nociceptive sensitivity for brief thermal stimuli, older adults exhibited significantly reduced tolerance to prolonged heat stimulation. In contrast, tolerance to tonic cold pain was comparable across age groups. This dissociation suggests that age‐related changes in nociceptive processing are not global but modality‐specific, and may depend on the physiological and regulatory systems activated by different types of noxious stimulation.

The observed reduction in tolerance to prolonged heat, but not cold stimulation, provides further insight into the selective effects of aging on pain processing. To date, only a limited number of studies have assessed tonic heat pain tolerance in older adults. Among these, Zhou et al. (2020) demonstrated an age‐related decline in tolerance to prolonged heat, a finding corroborated by our results. However, no prior study has directly contrasted tonic heat and cold pain within the same cohort across age groups, rendering the present evidence novel in demonstrating a modality‐specific preservation of cold pain tolerance with aging.

In our study, the absence of age‐related decline in cold pain tolerance contrasts with the findings of Walsh et al. (1989), who, using the Cold Pressor Test, reported reduced tolerance to cold pain in older adults. Methodological heterogeneity, particularly concerning the thermal medium and the extent of tissue exposure, likely contributes to this variability, as water immersion engages broader thermoregulatory and autonomic responses than the localized air‐based stimulation employed in the present study. These distinctions may underlie the absence of an age‐related decline in cold pain tolerance observed here. This absence of age‐related decline in cold pain tolerance contrasts with the findings of Walsh et al. (1989), who reported reduced tolerance to cold pain in older adults using the Cold Pressor Test (CPT). Methodological differences may explain this discrepancy, particularly the extent of the skin surface exposed to the cold stimulus. While our protocol involved exposure of only the hand, Walsh et al. included both the hand and forearm, possibly amplifying nociceptive input and thermoregulatory demands. Supporting this interpretation, Washington et al. (2000) reported no age‐related differences using a protocol similar to ours. Furthermore, differences in the thermal conductivity of the stimuli (cold water in CPT vs. cold air in our study) may also account for these divergent results, as water facilitates faster heat dissipation, thereby intensifying cold perception.

### 4.1 Physiological Specificities of Heat vs. Cold Pain

A first mechanistic interpretation of the modality‐specific effects observed in this study concerns differences in autonomic and vasomotor responses elicited by heat and cold stimulation. Tonic cold exposure is known to induce marked sympathetic activation, including peripheral vasoconstriction, elevated blood pressure, and increased heart rate, whereas prolonged heat stimulation elicits comparatively weaker autonomic adjustments (Streff et al. 2010). This dissociation indicates that thermal nociception does not activate a unitary regulatory system; instead, heat and cold pain engage partially distinct homeostatic and physiological pathways. Supporting this view, Devoize et al. (2016) showed that individual adaptability to prolonged noxious stimulation depends on modality‐specific autonomic profiles, with heat stimulation associated with greater interindividual variability in both cardiovascular and behavioural responses. This higher physiological variability may partially explain the increased vulnerability of older adults to prolonged heat pain relative to cold pain.

Building on this interpretation, one plausible mechanism for the selective decline in heat pain tolerance with aging involves alterations in vasomotor function. Prolonged heat stimulation normally induces local vasodilation, whereas cold exposure elicits vasoconstriction (Charkoudian 2003; Holowatz et al. 2010). With advancing age, vasoconstrictive responses remain relatively preserved (Thompson et al. 2005), while vasodilatory capacity becomes progressively impaired (Mayhan et al. 2008; Minson et al. 2002). As a consequence, older adults may be less able to dissipate heat during sustained thermal stimulation, leading to accelerated local warming, earlier recruitment of heat‐sensitive nociceptors, and a more rapid rise in perceived pain. This mechanism is consistent with the reduced tolerance to prolonged heat observed in the present study, whereas preserved vasoconstriction during cold exposure may help maintain tolerance to cold pain by limiting heat loss and modulating further nociceptive drive. Recent evidence supports this interpretation: the onset of pain during tonic heat stimulation coincides with a local vasodilatory reflex mediated by C‐fibre activation (Devanne et al. 2024), a response that appears attenuated with aging and may contribute to the selective vulnerability of older adults to heat pain.

One potential concern when interpreting these findings lies in the asymmetry of the thermal gradients required to elicit pain under heat and cold stimulation (approximately +10°C above baseline for heat versus −20°C below baseline for cold). This temperature‐gap asymmetry could suggest that the cold stimulus was inherently less intense and therefore less sensitive to age‐related effects. However, the absence of a modality effect in younger participants—both behaviorally and subjectively—argues against this interpretation, as heat and cold were perceived and tolerated as comparably intense in this group. Accordingly, the heat‐specific decline observed in older adults is unlikely to reflect a confound related solely to stimulus intensity.

Additional evidence supports the notion of modality‐specific processing in nociception. Studies have shown that adaptation to tonic pain is also modality dependent: repeated exposure enhances tolerance only to the same type of stimulus and does not generalise across thermal modalities (Polianskis et al. 2002). This finding indicates that heat and cold pain are at least partially governed by distinct nociceptive pathways and regulatory mechanisms. It is plausible that these mechanisms evolve differently with aging, further contributing to the selective impairment observed for prolonged heat stimulation.

Taken together, these findings suggest that age‐related impairments in vasodilatory function may underlie reduced tolerance to prolonged heat pain, offering a physiologically plausible explanation for the selective vulnerability observed in older adults, though this hypothesis requires further confirmation.

### 4.2 Endogenous Pain Modulation

Beyond peripheral mechanisms, age‐related changes in descending endogenous pain modulatory systems may also contribute to altered pain tolerance. These systems are often assessed experimentally through conditioned pain modulation (CPM), the human correlate of diffuse noxious inhibitory control, which reflects the capacity of one painful stimulus to reduce the perception of another. Numerous studies have shown that CPM efficiency decreases with age (Edwards et al. 2003; Riley et al. 2010; Yarnitsky 2015), supporting the hypothesis of impaired descending inhibitory function in older adults. However, CPM responses are not correlated with static pain measures such as pain thresholds or tolerance assessed with the Cold Pressor Test (Granot et al. 2008; Yarnitsky 2015; Yarnitsky et al. 2008). This indicates that while altered descending inhibition may contribute to reduced pain regulation in aging, it cannot directly explain the selective decline in heat pain tolerance observed in our study, and additional factors are likely involved in this alteration.

### 4.3 Affective Networks and Subjective Pain Responses

Analyses of subjective ratings suggest a differential influence of aging on the sensory–discriminative and affective dimensions of pain. Older adults reported significantly higher pain intensity during prolonged heat stimulation compared to younger adults, indicating an increased sensory perception of heat pain. In contrast, unpleasantness ratings only showed a non‐significant trend toward higher values in older adults, suggesting that the affective dimension contributed minimally to the observed age effect. This dissociation suggests the view that aging predominantly alters the sensory–discriminative aspects of prolonged heat pain, rather than enhancing its affective unpleasantness.

Paradoxically, neuroimaging studies have documented age‐related alterations in affective‐nociceptive networks, including reduced activation in the anterior cingulate cortex, insula, and prefrontal regions (Cole et al. 2010; Terrasa et al. 2021; van der Meulen et al. 2024). However, the behavioural manifestation of these neural changes appears relatively subtle in this study, possibly due to the so‐called “positivity effect” observed in aging, which reflects a shift toward attenuated attention to negative information (Mather 2012). In the present study, this effect may have contributed to preserved affective responses despite increased sensory intolerance to heat, although the positivity effect likely interacts with task demands, cognitive load, and attentional focus.

Taken together, these findings highlight the selective vulnerability of older adults to prolonged heat stimulation and underscore the importance of considering both the modality and duration of nociceptive stimuli when assessing age‐related changes in pain processing. They also suggest that age‐related changes in pain perception are not solely attributable to central modulation but may arise from a complex interplay between peripheral vascular reactivity, autonomic adaptation, and sensory processing.

### 4.4 Limitations

The study has several limitations. One is the relatively modest sample size, which may limit the detection of smaller effects and requires replication in larger and more diverse populations. The choice of air‐based thermal stimulation, while allowing for precise control and comparability between heat and cold modalities, limits direct comparison with the broader literature using water‐based paradigms. Additionally, although vasomotor mechanisms were discussed as potential contributors to the observed findings, this interpretation remains speculative in the absence of direct physiological measurements such as skin blood flow or autonomic responses. Future research combining nociceptive, vascular, and neurophysiological assessments would help clarify the respective contributions of peripheral and central mechanisms to age‐related differences in pain tolerance.

## 5 Conclusion

This study contributes to the growing body of evidence on how aging selectively affects pain processing, showing that older adults exhibit reduced tolerance to tonic heat stimulation, while tolerance to cold pain and heat pain tolerance thresholds are relatively preserved. These findings emphasize the need to consider both the modality and duration of nociceptive stimuli when assessing age‐related changes in pain perception. The selective decline in tonic heat pain highlights the complexity of pain regulation in older adults and underscores the importance of future multimodal investigations to disentangle these mechanisms.

## Author Contributions

The experiments were conducted by Julia Devanne and Louise Trocmet. The data were analysed by Julia Devanne, and the results were critically examined by Julia Devanne and Louise Trocmet. Julia Devanne was primarily responsible for drafting the manuscript. All authors have approved the final version of the manuscript and agree to be accountable for all aspects of the work.

## Funding

This work was funded by the French National Research Agency (ANR) through the Programme d'Investissement d'Avenir (ANR‐17‐EURE‐0022) and the Strasbourg Pain Initiative Consortium. This study has been published under the framework of the IdEx Unistra supported by the Investments for the Future program of the French Government.

## Conflicts of Interest

The authors declare no conflicts of interest.
